# Discovery of fusion circular RNAs in leukemia with *KMT2A::AFF1* rearrangements by the new software CircFusion

**DOI:** 10.1093/bib/bbac589

**Published:** 2022-12-30

**Authors:** Anna Dal Molin, Caterina Tretti Parenzan, Enrico Gaffo, Cristina Borin, Elena Boldrin, Lueder H Meyer, Geertruij te Kronnie, Silvia Bresolin, Stefania Bortoluzzi

**Affiliations:** Department of Molecular Medicine, University of Padova, Padova, Italy; Onco-Hematology, Stem Cell Transplant and Gene Therapy Laboratory, IRP-Istituto di Ricerca Pediatrica, Padova, Italy; Department of Molecular Medicine, University of Padova, Padova, Italy; Department of Molecular Medicine, University of Padova, Padova, Italy; Onco-Hematology, Stem Cell Transplant and Gene Therapy Laboratory, IRP-Istituto di Ricerca Pediatrica, Padova, Italy; Department of Pediatrics and Adolescent Medicine, Ulm University Medical Center, Ulm, Germany; Department of Molecular Medicine, University of Padova, Padova, Italy; Department of Biology, University of Padova, Padova, Italy; Department of Pediatrics and Adolescent Medicine, Ulm University Medical Center, Ulm, Germany; Department of Molecular Medicine, University of Padova, Padova, Italy; Department of Pediatrics and Adolescent Medicine, Ulm University Medical Center, Ulm, Germany; Department of Maternal and Child Health, University of Padova, Padova, Italy; Department of Molecular Medicine, University of Padova, Padova, Italy; Interdepartmental Research Center for Innovative Biotechnologies (CRIBI), University of Padova, Padova, Italy

**Keywords:** fusion circular RNA, fusion transcripts, chromosomal translocations, leukemia, KMT2A::AFF1

## Abstract

Chromosomal translocations in cancer genomes, key players in many types of cancers, generate chimeric proteins that drive oncogenesis. Genomes with chromosomal rearrangements can also produce fusion circular RNAs (f-circRNAs) by backsplicing of chimeric transcripts, as first shown in leukemias with *PML::RARα* and *KMT2A::MLLT3* translocations and later in solid cancers. F-circRNAs contribute to the oncogenic processes and reinforce the oncogenic activity of chimeric proteins. In leukemia with *KMT2A::AFF1* (*MLL::AF4*) fusions, we previously reported specific alterations of circRNA expression, but nothing was known about f-circRNAs. Due to the presence of two chimeric sequences, fusion and backsplice junctions, the identification of f-circRNAs with available tools is challenging, possibly resulting in the underestimation of this RNA species, especially when the breakpoint is not known. We developed CircFusion, a new software tool to detect linear fusion transcripts and f-circRNAs from RNA-seq data, both in samples for which the breakpoints are known and when the information about the joined exons is missing. CircFusion can detect linear and circular chimeric transcripts deriving from the main and reciprocal translocations also in the presence of multiple breakpoints, which are common in malignant cells. Benchmarking tests on simulated and real datasets of cancer samples with previously experimentally determined f-circRNAs showed that CircFusion provides reliable predictions and outperforms available methods for f-circRNA detection. We discovered and validated novel f-circRNAs in acute leukemia harboring *KMT2A::AFF1* rearrangements, leading the way to future functional studies aimed to unveil their role in this malignancy.

## Introduction

Circular RNAs (circRNAs) are stable RNA molecules generated by backsplicing, which juxtaposes a downstream 5′ donor splice site to an upstream 3′ splice acceptor site [1]. CircRNAs can regulate biological processes and drive cancer development and progression with different mechanisms, such as the interaction with microRNAs and RNA-binding proteins, and encoding specific peptides [2]. Dysregulation of circRNAs plays a role in solid cancer as well as in several hematological malignancies, including leukemias of the myeloid [3–8] and lymphoid lineages [9–12]. As stable molecules detectable in body fluids, circRNAs are regarded as ideal diagnostic and prognostic biomarkers [13, 14]. Plus, they can represent interesting new targets for therapeutic applications.

Chromosomal translocations harbored by cancer genomes are important oncogenic drivers. These rearrangements join parts of different genes often located on different chromosomes generating chimeric proteins that are well-known players in many types of malignancies, valid diagnostic markers and therapeutic targets, as exemplified by the paradigmatic case of *BCR::ABL1* leukemia [15].

Besides linear fusion transcripts, rearranged cancer genomes also express fusion-circRNAs (f-circRNAs), as reviewed in [1, 16, 17],. In acute promyelocytic leukemia (APL) with *PML::RARα* translocations and acute myeloid leukemia with *KMT2A::MLLT3* (*MLL::AF9*) fusions, f-circRNAs can be oncogenic or sustain the oncogenic properties of chimeric proteins [18]. In lung cancer, two f-circRNAs derived from *EML4::ALK* fusion have been identified, and proposed as a liquid biopsy biomarker [19, 20], whereas another f-circRNA from *SLC34A2::ROS1* translocation was proven to boost cancer cell migration [21]. In chronic myelogenous leukemia with *BCR::ABL1* fusion, the f-circRNA circBA9.3 was shown to promote proliferation and repress apoptosis by improving *BCR::ABL1* transcript translation or preventing its degradation [22]. As a proof of concept that f-circRNAs derive from genomes with rearrangements, cells engineered with *NPM1::ALK* fusion were proven to express *de novo* the same f-circRNAs found in patients [17].

The *KMT2A::AFF1* (*MLL::AF4*) translocation is common in patients with *MLL* rearranged acute lymphoblastic leukemia and marks an aggressive subtype of infant disease. Data about circRNA expression and role in this leukemia are still scanty. We previously showed that both *KMT2A* and *AFF1* genes each express several circular isoforms in normal hematopoiesis, whose expression can be affected by the rearrangement in leukemic cells [11], and the contribution to leukemogenesis of a circAFF1 isoform had been suggested [23]. However, nothing is known about f-circRNAs generated from the *KMT2A::AFF1* fusion. Therefore, the aim of this study is the development of a bioinformatic pipeline and its evaluation and application in case studies.

RNA sequencing (RNA-seq) approaches have allowed researchers to identify several fusions in cancer that were not detected by routine cytogenetic analyses [24]. Nevertheless, f-circRNA detection from RNA-seq data is not trivial since they include two sequences not present in the normal genome: the fusion junction, in which two genomic regions far apart in normal genomes are juxtaposed, and the backsplice junction, typical of circRNAs, connecting in reverse order two sequences of the fusion partner genes.

Although many tools have been developed to detect linear fusion transcripts, only Fcirc [25] and Acfs [26] can find f-circRNAs. Fcirc predicts f-circRNAs based on known fusions and reports only the transcripts coherent with a single fusion for each pair of translocation partner genes. This can be limiting when analyzing samples with complex rearrangements, with two or more breaks in the same chromosomal region and with non-corresponding reciprocal translocations, commonly found in cancer cells [27–30]. Acfs detects circRNAs and f-circRNAs, but not linear fusions, and hardly discriminates fusion junctions originating from gene family members with high sequence similarity [26].

We developed CircFusion, a bioinformatics tool to detect linear fusions and f-circRNAs from RNA-seq data, in samples in which the translocation breakpoint or only the fusion gene pair is known. Moreover, CircFusion can be run in a ‘discovery mode’ using a broad list of fusion partner gene pairs observed in diseases. CircFusion can also treat complex cases including multiple breakpoints and non-corresponding junctions in the first and second chromosome derivatives. Our tests on simulated and real data showed that CircFusion performs well in detecting f-circRNAs and linear fusion transcripts, representing an advance compared with current methods. Importantly, in this case study, we report the discovery with this software tool of new f-circRNAs in acute leukemia harboring *KMT2A::AFF1* rearrangements.

## Materials and methods

### Simulations of RNA-seq datasets

Two RNA-seq datasets were generated, containing reads of f-circRNAs and linear fusions from chimeric genes, to evaluate CircFusion ability to correctly detect and classify them. Eighteen pairs of genes were considered, arranged in 4 direct fusions (‘single breakpoint’), 4 reciprocal fusions (‘reciprocal’), 4 fusions joined genes with two breakpoints for each gene pair (‘multiple breakpoints’) and 6 fusions originated from pairs of paralog genes (‘paralog gene’). Furthermore, for 6 gene pairs, the fusion read junction mapped at the ends of the annotated exons (‘annotated exon junction’), while for 12, it mapped in the middle of the fused exons (‘not annotated exon junction’) (Supplementary Table S1).

Fusion, alternative fusion transcript and f-circRNA reads were simulated with coverage 4×, 2× and 2×, respectively. For each gene pair, one or two f-circRNAs were simulated, plus the fusion transcripts deriving from the direct and or the reciprocal translocation events, along with one or two additional linear fusions transcripts simulating products of alternative breakpoints. Overall, we simulated 40 reads encompassing the f-circRNA backsplices and 114 reads supporting the linear fusion transcripts (of which 72 for fusion transcripts and 42 for fusion alternative transcripts) (Supplementary Table S1). All read sequences were generated error-free, for both read lengths of 100 and 150 nt.

A dataset of B-cells from peripheral blood of a healthy donor (GEO series ID GSE110159), without any translocation expected, was used as negative control, to test CircFusion detection of false positive reads. First, CirComPara2 [31] was run to detect and quantify linear transcripts from the RNA-seq sample of B-cells. Then, linearly unmapped reads, i.e. reads that failed to be linearly aligned to the reference genome, were used as a background dataset for the simulation test, to which the simulated reads from the two aforementioned sets of fusions were added separately.

For the parameter tuning analysis, 12 different overlap values over the breakpoint junction (1 to 10, 15 and 20 nt), and 5 minimal numbers of matches (4, 5, 10, 15 and 20 matches) required in the alignment were tested.

**Figure 1:**
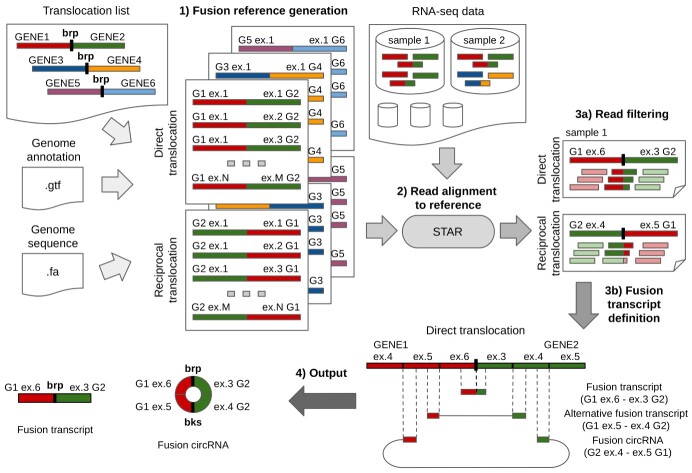
CircFusion workflow schematizing main analysis steps. For the translocation list given in input, a reference sequence set is generated using all combinations of gene exons and used as reference for read alignment using STAR, then alignments are scanned to detect the reads crossing the fusion junction, and to classify them in fusion transcripts, alternative fusion transcripts and f-circRNAs (brp, fusion breakpoint; bks, backsplice).

### RNA-seq datasets from samples with chromosomal rearrangements

RNA-seq datasets of cancer samples and cell lines bearing chromosomal translocations, in which f-circRNAs were previously identified and validated were downloaded from NCBI: 8 APL samples harboring the *PML::RARα* fusion (3 NB4 cell line and 5 patient samples; average sequencing depth of 49 million single-end reads) (BioProjectID PRJNA315254) [18], and 9 samples of lung adenocarcinoma H3122 cell line harboring the *EML4::ALK* fusion (average sequencing depth of 15 million pair-end reads) (BioProjectID PRJNA350335) [32] were downloaded from NCBI.

We further obtained RNA-seq data of the RS4;11 cell line (DSMZ), and five samples of patients with B-cell precursor acute lymphoblastic leukemia (BCP-ALL) carrying *KMT2A::AFF1* (*MLL::AF4*) rearrangements, including three patients and two patient-derived xenograft samples [33].

For each sample, high-depth RNA-seq data were obtained using total RNA extracted with TRIzol reagent (Thermo Fisher Scientific, Waltham, Massachusetts) and precipitated with isopropanol, with RNA integrity number (RIN) > 7, assessed with Agilent 2100 Bioanalyzer (Santa Clara, CA, USA). RNA libraries were prepared with the TruSeq Stranded Total RNA Ribo-Zero Gold kit and sequenced with an Illumina® HiSeq2000 (San Diego, CA, USA) at an average sequencing depth of 70 million reads per sample for patients (paired-end reads of 150 bp) and 400 million reads for the cell line. RNA-seq samples were analyzed through CirComPara2, and linearly unmapped reads for each sample were used by CircFusion to search for fusion RNAs.

Written informed consent was obtained from the parents or legal guardians of patients before sample collection and in accordance with the Declaration of Helsinki. The study approval was granted by institutional review committees at each participating center.

### Generation of the fusion reference sequence set and read alignment

Bedtools v2.28.0 [34] was used for file manipulation and exon sequence extraction for each fusion gene, as described in the Results section. R v4.1.2 and Bash programming languages were used to create the reference file in FASTA format with all the possible combinations between the exons of the fusion genes.

A reference sequence with exon combinations is generated for each translocation given in input (Figure 1), including, for the direct translocation, all the exon pairwise combinations, where the first exon belongs to the first gene in the pair and the second exon to the second gene, and, for the reciprocal translocation, all the combinations by inverting the gene order. The reference sequence files of all translocations are then collected into a single file.

A list of 305 fusion partner gene pairs observed in cancer, provided in the file ‘known_fusions.txt’ uploaded in GitHub, was collected from the Cosmic database.

The STAR aligner v2.7.5 [35] was used to align RNA-seq reads to the fusion reference sequence (See Supplementary Methods).

### Fusion transcript definition

The reads mapping on the reference sequence set and overlapping the direct or the reciprocal translocation junctions are considered for fusion transcript definition. An alignment of at least 50 nt, and, at the size of shorter overlap, of at least 5 nt and at most 17% of mismatches, was required.

Then, for both the direct and the reciprocal translocations, the reads are classified in fusion transcripts and f-circRNAs, based on the exons of the reference sequences where they map (Figure 1). Moreover, additional fusion transcripts from breakpoints different from those indicated by the user are classified as ‘alternative fusion transcripts’.

### Performance evaluation

CircFusion performance was evaluated using precision $\big(\mathrm{TP}/\big(\mathrm{TP}+\mathrm{FP}\big)\big)$, recall $\big(\mathrm{TP}/\big(\mathrm{TP}+\mathrm{FN}\big)\big)$, and F_1_ score ($2\ast \big(\big(\mathrm{recall}\ast \mathrm{precision}\big)/\big(\mathrm{recall}+\mathrm{precision}\big)\big)$), where TP (true positive) is the number of linear fusion transcripts or f-circRNAs correctly predicted by the algorithm, FP (false positive) is the number of the predicted fusion transcripts that are not true and FN (false negative) is the number of true fusion transcripts that were not detected.

The execution time of CircFusion was measured in real time (minutes).

### F-circRNAs validation

RNA was treated with RNAse R (Epicentre) using 2 U/μg of enzyme/RNA, mixed with RNase R buffer and incubated at 37°C, then purified using purification columns of Zymo kit (Zymo Research). Treated RNA (150 ng) was retro-transcribed using random hexamers (Invitrogen™, Thermo Fisher Scientific, Waltham, Massachusett) and SuperScript II Reverse Transcriptase (Invitrogen™, Thermo Fisher Scientific).

PCR reactions from RS4;11 cDNA were carried out for 42 cycles using Taq Polymerase I and Buffer 10X (Roche), dNTPs (GE Healthcare) and custom primers (IDT, Integrated DNA Technologies; KMT2A_FW_ex9–10 CTTTAAGGAGGATTGTGAAGCAG; AFF1_REV_ex2 CCTGGTTGCGTCTTTCCTTC) of 20 μM final concentration. Primers to validate the backsplice junction of circRNAs were designed to be ‘divergent’ in the corresponding genomic exons and to become ‘convergent’ with the final amplification of the backsplicing. PCR products were purified using Illustra ExoProStar enzymes (Cytiva, USA). Sanger sequencing employing the Applied Biosystem Genetic Analyzer 3500 DX was used to confirm the backsplice and the fusion junction sequences.

### Graphical visualization

R version 4.2.1 was used to generate the figure plots. The R package ggplot2 v3.3.6 [36] was used for bar plots and line plots, whereas the circlize v0.4.15 [37] package was used for circular plots.

## Results

### The CircFusion workflow

CircFusion is a new bioinformatics tool to detect f-circRNAs and linear fusion transcripts from RNA-seq data and is freely available on GitHub (https://github.com/annadalmolin/CircFusion) and DockerHub (https://hub.docker.com/repository/docker/annadalmolin/CircFusion). The software is written in Bash and R programming languages, allows full customization of the parameters for read alignment and fusion transcript definition, and leverages parallel computing to save computational time.

CircFusion takes as input the genome sequence in FASTA format, the gene annotation file in GTF format, the list of genomic translocations of interest (i.e. the two genes involved and, optionally, the exons flanking the direct and the reciprocal translocation junction and thus expected to be joined in fusion transcripts) and the RNA-seq reads in FASTQ format. A HTML summary file and the list as CSV table and plain text of f-circRNAs and linear fusions detected, along with additional information about read alignments supporting the detected fusion transcripts, are given in the output.

The pipeline consists of three main steps: (i) generation of reference sequences for the translocation junctions given in input; (ii) alignment of the RNA-seq reads to the reference sequences; (iii) detection of linear fusion and f-circRNA transcripts from the alignments (Figure 1).

At first, a reference sequence set is generated with all the translocations in the input file, joining in all the pairwise combinations both the exons of the first to the exons of the second gene (‘direct’ reference), and inverting the gene order (‘reciprocal’). CircFusion can be run searching for one or more translocations of interest, or in a ‘discovery mode’ extending the search to a larger set of fusion partner gene pairs observed in cancer. The user can benefit from the provided file ‘known_fusions.txt’ in the GitHub page of CircFusion (see Data Availability) collecting a large number of known fusions in cancer, or by customizing the file by adding or removing fusion gene pairs.

In the second step, the RNA-seq reads of each sample are aligned on the reference sequence set through the STAR aligner with optimized settings, as specified in Methods section. The alignments are then scanned to detect the reads crossing the fusion junction, for each translocation gene pair, and to classify them according to the provided input, as follows:

(i) When only the gene pair is specified in the input, without information on the fused exons, then the complete reference sequence set is scanned;
(ii) If the exons fused in both derivatives are given in input, then CircFusion uses them to define fusion transcripts;
(iii) When only the exons of the direct fusion are indicated, the corresponding reciprocal translocation is considered to retrieve the exons of the reciprocal compatible with the fusion.

The reads mapping on the fusion junction define ‘fusion transcripts’, and read alignments indicating a backsplice on the fusion transcript define ‘f-circRNAs’ (Figure 1). The reads mapped on the fusion transcript in paired exons other than those indicated as the fusion junction exons are labeled as ‘alternative fusion transcripts’, which can denote additional translocations in the sample. In this case, the user can run the analysis again, indicating these newly discovered translocations, to search for further f-circRNAs compatible with the newly discovered junctions.

The output includes an HTML report with the f-circRNAs and linear RNAs identified, summarized for each translocation. Plain text and CSV files for each sample report the genomic coordinates of the fusion translocations, also of the backsplice for f-circRNAs, and the number of fusion supporting reads. In addition, for each translocation and for each sample analyzed, information is given for each fusion read, specifying the genes and the exons involved in the translocation and other details about the read alignment. Furthermore, CircFusion provides two plain text report files for each translocation, with the number of supporting reads and the number of fusions detected for each type, respectively.

**Figure 2:**
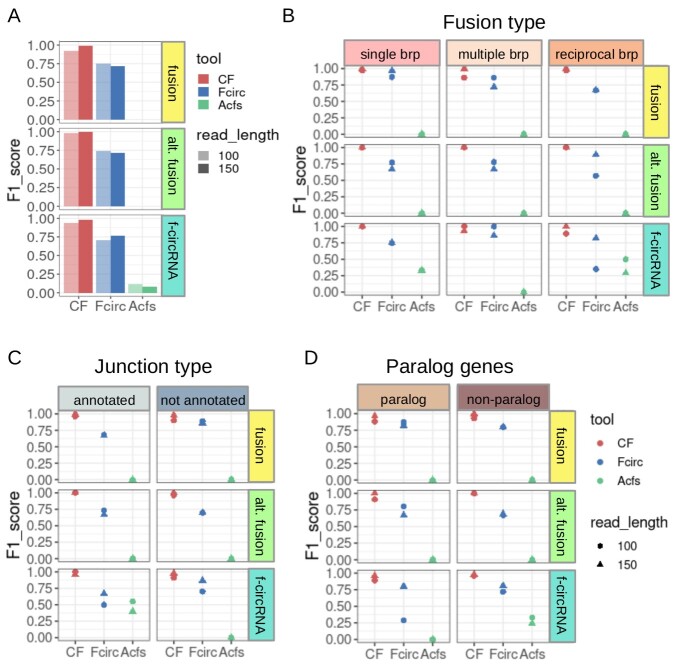
CircFusion, Fcirc and Acfs performances in linear and circular fusion transcript detection from simulated RNA-seq data. (**A**) F1 score of CircFusion (CF, red), Fcirc (blue) and Acfs (green); light and dark colors are used for 100 and 150 nt reads. The F1 score achieved by the tree tools is shown separately by (**B**) fusion type, (**C**) junction type and (**D**) in presence or absence of translocations involving paralog genes (dot colors represent different tools, as in panel A; brp, breakpoint; fusion, fusion transcript; alt. fusion, alternative fusion transcript).

### Simulation of f-circRNAs and linear fusions in RNA-seq data

The ability of CircFusion to accurately detect and classify, in simulated RNA-seq data, f-circRNAs and fusion transcripts was tested and compared with the similar purpose tools Fcirc [25] and Acfs [26].

We simulated two RNA-seq read datasets (with reads 100 and 150 bases long, respectively) containing sequences derived from f-circRNAs and linear fusion RNAs compatible with translocation events under different scenarios (Supplementary Table S1 and Methods). In particular, we considered three types of fusion reads, derived from: (i) the direct translocation (‘single breakpoint’), (ii) the reciprocal translocation and (iii) multiple breakpoints for the same pair of genes (‘multiple breakpoints’). Plus, fusion reads derived from pairs of genes paralog to those given in input. Finally, since we observed in real data that the fusion junction does not always fit with the annotated exon junctions, in our simulations, we mixed reads that encompass annotated exon junctions and mapping within exon boundaries, i.e. on ‘not annotated’ junctions.

Simulated data were based on rearrangements reported in the literature. For instance, the *SLC34A2::ROS1* translocation [21] represents complex rearrangements in which the same pair of genes is joined with multiple breakpoints. Moreover, the *BCR::ABL1* [38] and *NSMCE2::PVT1* [39] fusions were included along with their reciprocal translocations (*ABL1::BCR* and *PVT1::NSMCE2*, respectively). Reads from paralog genes (i.e. *KMT2B, MLLT3* and *MLLT10* genes, paralogs of *KMT2A, MLLT1* and *MLLT6*, respectively) were added to the read-set to test the tool capability in discriminating them, in a more challenging scenario.

### CircFusion correctly detects f-circRNAs in simulated data

Test on simulated data validated CircFusion algorithm, showing as well that it is an improvement over Fcirc and Acfs (Figure 2A and Supplementary Figure S1A).

CircFusion achieved high precision and recall, correctly identifying all the fusion reads without false positives in 5 cases out of 12, and scoring close to one in the others (Supplementary Figure S1A).

Both in the detection of f-circRNAs and linear fusion transcripts, CircFusion F_1_ score was high, performing better compared with Fcirc (range 0.94–1 versus 0.71–0.77). Moreover, both methods surpassed Acfs (range 0–0.12) (Figure 2A). Also considering the results obtained in the different scenarios separately, CircFusion performed better than the other methods, in most cases (Figure 2B–D and Supplementary Figure S1B–D). Regarding the three fusion types (single breakpoint, multiple breakpoints and presence of a reciprocal translocation), the major difference between methods was observed for recall and F_1_ scores, underlying the low rate of false negatives of CircFusion (Figure 2B and Supplementary Figure S1B). In the case of a single breakpoint, both for the alternative fusion and f-circRNA detection, and for both read lengths, the recall and F1 score of CircFusion were maximal among the three methods. With multiple breakpoints, CircFusion recall and F_1_ score remained maximal in most cases, while Fcirc varied more, and Acfs performed worse. The gap between the CircFusion and Fcirc was more evident in the presence of reciprocal translocations and for f-circRNA detection. Regarding the junction type, the performances were as above, with main improvements of CircFusion applied to f-circRNA discovery (Figure 2C and Supplementary Figure S1C). Finally, CircFusion performed better also in the scenario with confounding reads derived from paralog genes, particularly with longer reads when searching for f-circRNAs (Figure 2D and Supplementary Figure S1D).

In the parameter tuning analysis, we tested CircFusion using 60 combinations of values for minimum read overlap over the backsplice junction and number of matches required in the alignment, to assess the performance of the tool (Supplementary Figure S2). For both linear fusions and f-circRNAs, the precision increased with stringency, already exceeding 0.8 with an overlap of at least 4 nt. The F_1_ score increased for f-circRNA detection when raising both tested parameters, whereas it slightly decreased for fusion transcript detection. Thus, we set as default an overlap of 5 nt, to reduce almost the 90% of FP, and a minimum number of matches of 5, to reduce about the 90% of FP and find at least the 80% of TP.

Regarding computational efficiency, CircFusion spent 17 s for each translocation to generate the reference sequence file. Then, it took 7.46 and 7.50 min analyzing the 100 and 150 nt read datasets (4.9 million reads, 8 processors), resulting faster than Acfs (10.19 and 10.27 min) and slower than Fcirc (4.22 and 4.13 min). The better performance of CircFusion is, therefore, associated with a slightly increased computational time, compared with Fcirc.

### CircFusion efficiently detects known f-circRNAs in RNA-seq data of cancer samples with chromosomal rearrangements

We next tested CircFusion on RNA-seq data of cancer cells expressing f-circRNAs and linear fusion transcripts that were previously identified by PCR-based techniques and experimentally validated: 8 APL samples harboring the *PML::RARα* fusion (3 NB4 cell line and 5 patient samples) [18], and 9 samples of lung adenocarcinoma H3122 cell line with the *EML4::ALK* fusion [32].

Both the APL and H3122 RNA-seq data have been used in the publication presenting the Fcirc method [26], whereas Acfs was tested only on the APL data [25]. Thus, we could compare the new results obtained by the CircFusion analysis of APL data with those previously obtained by both Fcirc and Acfs, whereas the results of the H3122 cell line only with Fcirc.

**Figure 3:**
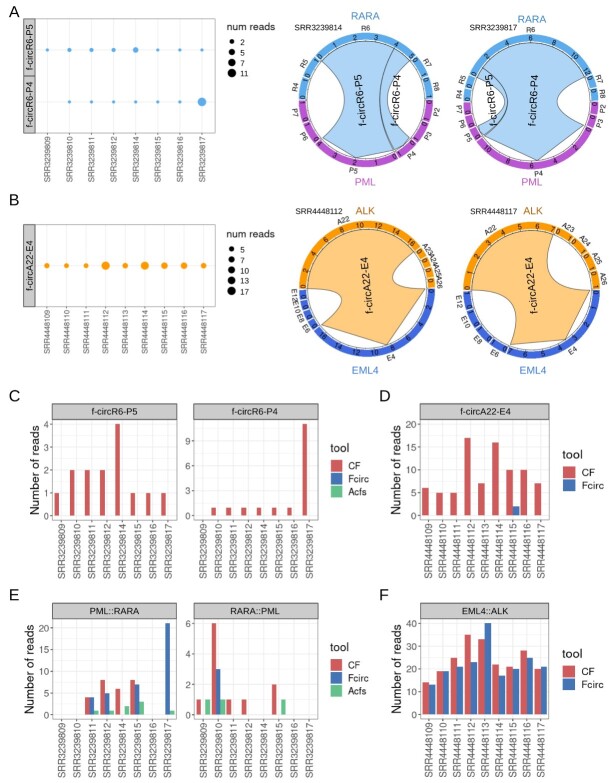
Detection, by CircFusion, Fcirc and Acfs, of known f-circRNAs from RNA-seq datasets of cancer cell lines and patient samples with chromosomal rearrangements. Number of reads supporting the identification, by CircFusion, of the f-circRNAs previously validated: (**A**) f-circR6-P5 (joining *RARα* ex.6 to *PML* ex.5; 4 nt overlap over the fusion junction) and f-circR6-P4 (joining RARα ex.6 to *PML* ex.4; 2 nt overlap) in the *PML::RARα* dataset, and (**B**) f-circA22-E4 (joining *ALK* ex.22 to *EML4* ex.4) in *EML4::ALK* samples. The circos plots on the right show the f-circRNA exons involved in the fusion and the read count supporting the f-circRNA chimeric backsplice junction, in two samples of each dataset (the border sectors represent the exons, identified by the first letter of the gene and the exon number, the numbers inside the sectors give the number of reads mapping on each exon, the belts represent f-circRNAs and are colored as the first gene in the fusion, with the pointed end indicating the second gene). Supporting reads detected by different methods for the validated f-circRNAs (**C** and **D**) and linear fusion transcripts (**E** and **F**) in the *PML::RARα* (**C** and **E**) and in the *EML4::ALK* (**D** and **F**) samples.

In APL samples, CircFusion successfully identified both previously validated *PML::RARα* f-circRNAs [18], supported by from 1 to 11 reads (average sequencing depth of 49 M reads). FcircR6-P5 (joining exon 6 of *RARα* with exon 5 of *PML*) and f-circR6-P4 were found, respectively, in all and in all but one samples (Figure 3A). Moreover, in all the H3122 lung cancer cell line samples bearing the *EML4::ALK* fusion, CircFusion detected f-circA22-E4 [19, 20] (5–17 reads per sample, average dataset depth of 15 M read pairs; Figure 3B).

CircFusion spent 80 min on average per sample (40 CPUs) when analyzing the *PML::RARA* dataset, and 200 min for the *EML4::ALK* dataset. The main difference between the two execution times is due to the data structure: while the first dataset has single-end reads, the second is paired-ended. By tuning the CircFusion parameters, the execution time can decrease, but with the risk of worsening the performance.

The number of samples in which CircFusion, Fcirc and Acfs identified the three known f-circRNAs, with the corresponding numbers of supporting reads, is shown in Figure 3C and D. Fcirc detected different f-circRNA isoforms in one sample of the *PML::RARα* dataset, but none of the two validated, whereas Acfs detected no f-circRNAs at all (Figure 3C). Conversely, in the *EML4::ALK* dataset, Fcirc found the validated f-circA22-E4 in one sample (Figure 3D). Considering the linear fusions in the two datasets, the same data are shown in Figure 3E and F**.** CircFusion successfully identified the *PML::RARα* and/or *RARα::PML* fusions in all but two of the eight samples. In the *EML4::ALK* dataset, CircFusion identified the fusion breakpoint in all samples concordantly with Fcirc, and no evidence was found of transcripts from a reciprocal translocation, in line with literature data [40].

### Discovery of f-circRNAs in acute leukemia samples with *KMT2A::AFF1* translocation

Using CircFusion, we identified for the first time f-circRNAs in acute lymphoblastic leukemia with *KMT2A::AFF1* translocation.

**Figure 4:**
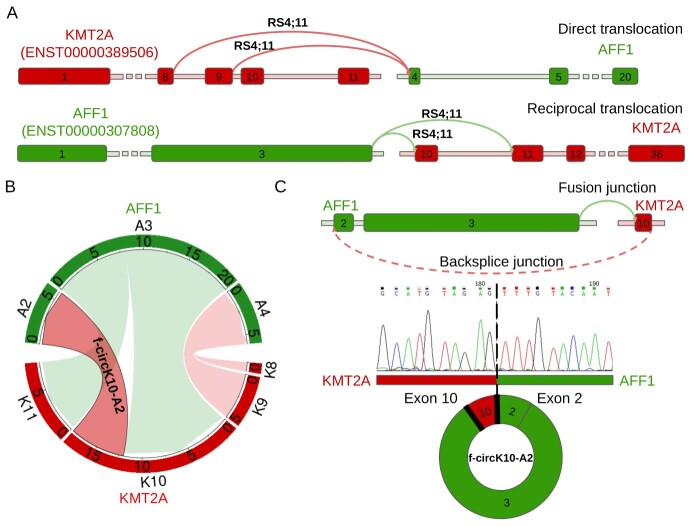
F-circRNA and linear fusion transcripts expressed by the RS4;11 cell line with *KMT2A::AFF1* translocations and validation of F-circK10-A2. (**A**) The read and green colored boxes represent *KMT2A* and *AFF1* exons, respectively, and the arcs show the direct and reciprocal fusions detected by PCR and Sanger sequencing, with the arc color indicating the first gene in the fusion; (**B**) linear fusion transcripts and f-circRNA detected by CircFusion in the RS4;11 cell line; the circos plot border sectors show the exons joined by the fusion (fusion transcripts, light colors) and backspliced (f-circRNAs, dark color with black border), the thickness of the belts shows the read count supporting each transcript (number of supporting reads normalized over 10 M of linearly unmapped reads), and belt color indicates the first gene in the fusion. (**C**) F-circK10-A2 validation by Sanger sequencing, confirming the chimeric backsplice junction joining *KMT2A* exon 10 with *AFF1* exon 2.

Initially, we focused on the RS4;11 cell line carrying multiple direct *KMT2A::AFF1* (K-A) and reciprocal *AFF1::KMT2A* (A-K) fusions, concordantly detected by PCR and Sanger sequencing (Figure 4A and Supplementary Table S2) and by CircFusion analysis (Figure 4B). We identified four linear fusion transcripts, likewise K8-A4 (joining *KMT2A* exon 8 to *AFF1* exon 4) and K9-A4 from the *KMT2A*-derivative, and A3-K10 and A3-K11 from the *AFF1*-derivative. Due to the different depth of the dataset carrying the *KMT2A::AFF1* fusion, to better compare the findings in the different samples, we considered the fusion supported reads as normalized over 10 M linearly unmapped reads (NR). Thus, the reciprocal fusion transcript A3-K10 was supported by 11.6 NR, whereas 0.86 NR were associated with the less expressed transcript (K8-A4) (Figure 4B).

Importantly, CircFusion detected an f-circRNA generated by backsplicing of exon 10 of *KMT2A* to exon 2 of *AFF1* (f-circK10-A2) in RS4;11 RNAse treated RNA (Figure 4B), expressed at a level (6.03 NR) comparable with those of the linear chimeric transcripts. Of note, the backsplice and the fusion junctions of f-circK10-A2 were confirmed by PCR with divergent primers placed on *KMT2A* exon 9–10 (FW) and *AFF1* exon 2 (RW) treated with RNAse R, followed by Sanger sequencing (Figure 4C).

**Figure 5:**
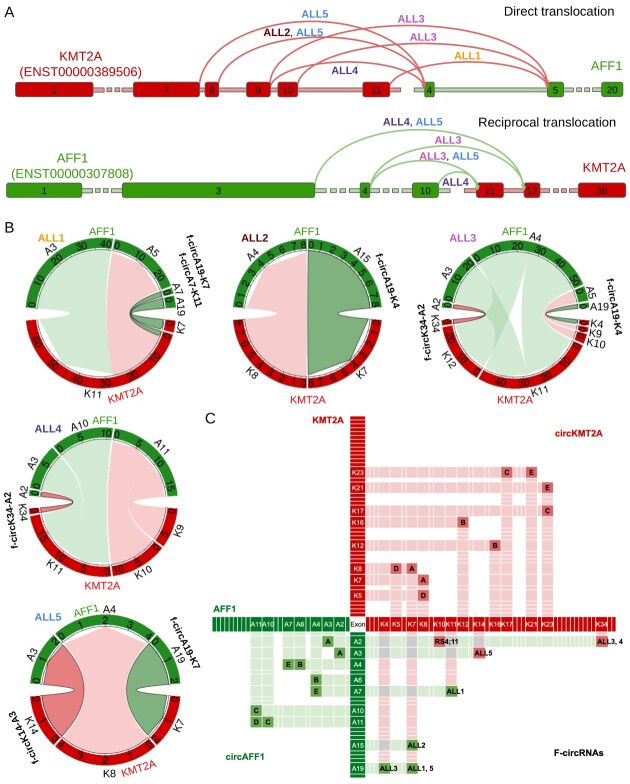
F-circRNAs and linear fusion transcripts detected in samples of BCP-ALL patients with *KMT2A::AFF1* translocations. (**A**) The read and green colored boxes represent exons, respectively, and the arcs show the breakpoint translocations detected by PCR, with the arc color indicating the first gene in the fusion; (**B**) linear fusion transcripts (light colors) and f-circRNAs (dark colors with black border) detected in patients by CircFusion (plot legend as in Figure 4**B**); (**C**) *KMT2A* and *AFF1* exons backspliced to generate canonical circRNAs expressed in blood cells (letters A-E identify circKMT2A and circAFF1 isoforms as in [11]) and chimeric f-circRNAs detected in patients and in the RS4;11 cell line with *KMT2A::AFF1* fusions (colors as in panels **A** and **B**).

The discovery of an f-circRNA in the *KMT2A::AFF1* cell line incited further investigation in patients. Thus, we collected five patient and patient-derived samples bearing different *KMT2A::AFF1* translocations (Figure 5A and Supplementary Table S2) [41] and profiled them by RNA-seq followed by CircFusion analysis. Multiple direct and reciprocal fusions were found (Figure 5B) and then confirmed by PCR.

It is worth noting that f-circRNAs were discovered in all the patient samples (Figure 5B and Supplementary Table S3), with a total of six distinct f-circRNAs detected, two derived from the K-A and 4 from the A-K translocation.

Two fusion circRNAs were recurrently detected in patients, f-circA19-K7 in ALL1 and ALL5, and f-circK34-A2 in ALL3 and ALL4. Three patients expressed two f-circRNAs each, with f-circA19-K7 coexisting with f-circA7-K11 in ALL1 and with f-circK14-A3 in ALL5, and ALL3 expressing both f-circK34-A2 and f-circA19-K4. In ALL1 and ALL5, the exons flanking the fusion junction (i.e. A3 and K7) were used in f-circRNAs (f-circK14-A3 and f-circA19-K7), while in the remaining cases, the circularized exons were more apart from the exons involved in fusion junctions.

Regarding the expression level of the discovered f-circRNAs, in ALL2, f-circA15-K7 was as abundant as the linear fusion transcript (linear junction K8-A4); in ALL5, both f-circRNAs detected were expressed at 50% of the linear fusion RNA, whereas in the other samples, the f-circRNAs were less expressed than linear transcripts (Figure 5B).

Considering the f-circRNAs produced by leukemia cells found by the present study and the circKMT2A and circAFF1 isoforms expressed in normal blood cells according to [11], we observed that half of the exons used in ‘aberrant chimeric’ f-circRNAs were commonly backspliced also in ‘canonical’ circRNAs (Figure 5C)*. KMT2A* exon 7, found in the recurrent f-circA19-K7 and in the most abundant f-circA15-K7, *AFF1* exon 2, backspliced in the recurrent f-circK34-A2, and in the f-circK10-A2 detected in the RS4;11 cell line, and *AFF1* exon 7 found in the f-circA7-K11, were also used in canonical circRNAs. In particular, *KMT2A* exon 7 was expressed in circKMT2A_A(K8-K7), previously shown to be the most abundantly expressed from the *KMT2A* gene, *AFF1* exon 2 was included in the very highly expressed circAFF1_A(A3-A2), and *AFF1* exon 7 was part of the less expressed circAFF1_E(A7-A4). Of note, *AFF1* exon 3, backspliced in the f-circK14-A3, was also used in circAFF1_A(A3-A2), joined through a splicing junction in 3′ instead of 5′.

In BCP-ALL, f-circRNAs expression levels were comparable with those of canonical circKMT2A and circAFF1 isoforms, when excluding circAFF1_A(A3-A2), which was far more abundant of all the other circRNAs in most samples (Supplementary Figure S3).

## Discussion

This study presents two highlights: the definition of the landscape of f-circRNAs in BCP-ALL with *KMT2A::AFF1* translocations and the development of CircFusion, a new software tool to identify fusion linear and circular RNAs from RNA-seq data performing better than state-of-the-art tools and that can have broad applications.

CircFusion has several advantages over the few similar existing tools. It can search for f-circRNAs both in samples where the exact fusion junction breakpoint is known and when only the two partner genes are defined, scanning all exon pair combinations to detect f-circRNAs compatible with any breakpoints. Importantly, CircFusion can manage complex rearrangements, which are commonly found in cancer cells, such as fusions with two or more breakpoints and non-corresponding reciprocal translocations in the first and second chromosome derivatives. Plus, in the ‘discovery mode’, CircFusion search can be broadened to a large set of fusion partner gene pairs customizable by the user.

The default parameters were optimized, but the analysis stringency can be conducted by the user, tuning the parameters for read alignment (f.i. the number of multiple alignments per read, and the minimum number of matches) and for fusion transcript definition (f.i. the number of mismatches allowed in the alignment, and the minimum base overlap on the fusion junction).

Notably, we demonstrated that CircFusion correctly identifies f-circRNAs and fusion transcripts from RNA-seq data with tests on simulated datasets containing known chimeric transcripts and by the reanalysis of samples, including leukemia and solid tumors bearing chromosomal rearrangements, in which f-circRNAs were previously detected and validated. For both linear fusion transcript and f-circRNA detection, in the simulation test, CircFusion had a lower rate of both false positives and false negatives than Fcirc and, especially, Acfs. Challenging the methods in scenarios different for fusion type, junction type, and presence of reads coming from translocations involving paralog genes revealed precision values comparable with Fcirc, and superior recall and F_1_ scores in most cases, underlying the low rate of false negatives of CircFusion. Moreover, when tested on real RNA-seq datasets of cancer cells bearing chromosomal translocations, CircFusion successfully identified all three previously experimentally validated f-circRNAs. Collectively, the benchmarking tests indicated that CircFusion can be a useful and performing tool to identify f-circRNAs from RNA-seq data. Some of the previously validated f-circRNAs were supported by a low number of reads. Thus, we recommend to consider that a low depth (e.g. lower than 10 M reads) would hamper the identification of weakly expressed transcripts, and to tune the stringency of the search parameters according to the sequencing depth.

The second highlight of this study came from the investigation of leukemia with the *KMT2A::AFF1* (*MLL::AF4*) fusion. We focused on this translocation since it marks an aggressive subtype of infant acute lymphoblastic leukemia with a still poor outcome. Data about circRNA expression and role in this particular disease is very limited [23, 42]. After the breakthrough discovery of oncogenic f-circRNAs in leukemia with *KMT2A::MLLT3* (*MLL::AF9*) [18], followed by the finding of f-circRNAs in other malignancies with different translocations [19–22], no data emerged about chimeric circRNAs in leukemias with other rearrangements involving the *KMT2A* gene.

By RNA-seq profiling and CircFusion analysis, we identified f-circRNAs produced by *KMT2A::AFF1* and cognate reciprocal fusions in all the samples analyzed, including the RS4;11 cell line and specimens of pediatric patients. The RS4;11 cell line bears multiple breakpoints, with four fusion transcripts detected. The breakpoint picture was in line with literature data for which the direct breakpoints in RS4;11 cell line have been shown to span between exon 8 and 9 of *KMT2A* and between exon 4 and 5 of *AFF1* [43, 44], and two reciprocal transcripts were identified [45]. Also in patients with *KMT2A::AFF1* fusions, up to 4 different breakpoints were present in most cases.

Of importance, f-circRNAs were discovered in all the leukemia samples analyzed, both in the RS4;11 cell line and in the patient specimens. In three out of five cases, we found two different f-circRNAs each. Two fusion circRNAs, f-circA19-K7 and f-circK34-A2, were recurrently detected in two cases. Of the 6 distinct f-circRNAs identified in patients, 4 were expressed from the *AFF1*-derivative and 2 were expressed from the *KMT2A*-derivative, as the f-circK10-A2 detected and validated in the RS4;11 cells. Considering all the samples and the cell line together, the expression of f-circRNAs and linear fusion transcripts was comparable in half of the cases, with the f-circRNAs being less abundant in the others. The f-circK10-A2 detected in RS4;11 cell line was not represented in patients, but the exon A2 was backspliced to generate the recurrent f-circK34-A2 in two patients (ALL3–4). We previously showed that, in normal haematopoiesis, multiple ‘canonical’ circRNAs are expressed by both *KMT2A* and *AFF1* genes [11]. In BCP-ALL, f-circRNA expression was lower than the very abundant canonical circAFF1_A(A3-A2), but comparable with that of the remaining several canonical circKMT2A and circAFF1 isoforms. Of note, specific exons (*KMT2A* exon 7, and *AFF1* exons 2 and 7) backspliced to generate the most abundant circRNAs of these genes in normal blood cells are also used in f-circRNAs produced by leukemia cells with rearranged genomes.

In conclusion, we propose a new, useful and effective software tool to define circular and linear fusion transcripts in cancer cells with rearranged genomes, and with our case study, we provide new data about f-circRNA expression in BCP-ALL with *KMT2A::AFF1* rearrangements and highlight new aberrant transcripts that can be searched for in other possibly larger patient cohorts. The discovery of f-circRNAs derived from pathognomonic *KMT2A::AFF1* fusions in infant ALL incites further investigation into their possible functional role in the future.

**Key Points**

- We defined the landscape of aberrant fusion circular RNAs in infant acute lymphoblastic leukemia with *KMT2A::AFF1* translocations.
- CircFusion is a new and useful software tool to efficiently detect linear and circular fusion transcripts from RNA-seq data.
- Tests on simulated and real RNA-seq data showed that CircFusion performs better than the few similar available tools.

## Supplementary Material

Fusion_paper_Supplementary-rev1_bbac589

## Data Availability

CircFusion tool is available on GitHub at https://github.com/annadalmolin/CircFusion and on DockerHub at https://hub.docker.com/repository/docker/annadalmolin/circfusion.

## Notes

**Anna Dal Molin** is a post-doc at the Computational Genomics Laboratory at the Department of Molecular Medicine, University of Padova. Her research interests include bioinformatics and transcriptomics, circular RNAs in leukemias and development of computational methods for circular RNA function prediction and characterization.

**Caterina Tretti Parenzan** is a PhD student in Pediatric Oncoematology of the University of Padua. Her research is mostly focused on the study of the oncogenic molecular mechanisms involving circRNAs in pediatric acute lymphoblastic leukemia.

**Enrico Gaffo** is post-doc at the Computational Genomics Laboratory at the Department of Molecular Medicine, University of Padova. His research interests include circular RNA, microRNA, advanced methods for RNA-seq data analysis and bioinformatics applied to cancer research.

**Cristina Borin** graduated in the master degree in Molecular Biology at the University of Padova at the Pediatric Hematology, Oncology and Hematopoietic cell&gene therapy laboratory of the IRP (Research Pediatric Institute), studying circRNAs in Acute Lymphoblastic Leukemia.

**Elena Boldrin** graduated within a joint PhD program between Ulm University Medical Center and Padua University, working on microRNAs and circular RNAs in B cell precursor acute lymphoblastic leukemia. She is now a postdoctoral fellow at Lund University in the department of Laboratory Medicine. Her main research interests focus on the impact of early life specific molecular determinants on B cell differentiation and on the development of pediatric malignancies.

**Lüder Hinrich Meyer** is a pediatric hemato-oncologist and associate professor at the Department of Pediatrics and Adolescent Medicine at Ulm University Medical Center, Germany. His research focusses on childhood lymphoblastic leukemia with a particular interest in leukemia initiation, development, biology, and translational aspects including identification of target structures for novel directed therapies.

**Geertruij te Kronnie** graduated at the University of Amsterdam in Medical Biology working on animal models of early development and disease. During the past 22 years she worked at the departments of Women’s and Children’s Health and Molecular Medicine of the University of Padova where she initiated large collaborative studies in pediatric leukemia research with a focus on transcriptomics of diagnostically relevant leukemia subtypes and more recently on germline risk variants of pediatric leukemia and cancer risk in general.

**Silvia Bresolin** is an assistant professor of Molecular Biology at the Department of Women and Child Health Department of the University of Padova. Her field of interest is focused on pediatric leukemia transcriptomics and genomics alterations, as well as functional genomics and development of novel disease models.

**Stefania Bortoluzzi** is an associate professor of Applied Biology at the Department of Molecular Medicine of the University of Padova, where she leads the Computational Genomics Laboratory. Her research interests include cancer genomics and transcriptomics, bioinformatics, systems biology, non-coding RNAs, circular RNAs, exosomal RNAs and hematologic malignancies.
