# Supplementary material for: Discovery of fusion circular RNAs in leukemia with KMT2A::AFF1 rearrangements by the new software CircFusion
Source: Brief Bioinform. 2022 Dec 30;24(1):bbac589. doi: 10.1093/bib/bbac589 (PMC9851293; doi:10.1093/bib/bbac589)
Supplement: Fusion_paper_Supplementary-rev1_bbac589 [file fusion_paper_supplementary-rev1_bbac589.pdf]

Discovery of fusion circular RNAs in leukemia with *KMT2A::AFF1* rearrangements by the new software CircFusion

Anna Dal Molin[1,&,*], Caterina Tretti Parenzan[2,&], Enrico Gaffo[1], Cristina Borin[1,2], Elena Boldrin[3,1,4,], Lueder H. Meyer[3], Geertruij te Kronnie[1], Silvia Bresolin[3,5,$], Stefania Bortoluzzi[1,6,$,*]

[1] Department of Molecular Medicine, University of Padova, Padova, Italy

[2] Onco-Hematology, Stem Cell Transplant and Gene Therapy Laboratory, IRP-Istituto di Ricerca Pediatrica, Padova, Italy

[3] Department of Pediatrics and Adolescent Medicine, Ulm University Medical Center, Ulm, Germany.

[4] Department of Biology, University of Padova, Padova, Italy

[5] Department of Maternal and Child Health, University of Padova, Padova, Italy

[6] Interdepartmental Research Center for Innovative Biotechnologies (CRIBI), University of Padova, Padova, Italy

[&] Equally contributing first authors

[$] Equally contributing last authors

[*] Corresponding authors

Supplementary Materials

Supplementary Methods **1**

Read alignment to the reference sequences 1

Supplementary Figures **2**

Supplementary Figure 1. Precision and recall in simulated RNA-seq datasets of CircFusion, Fcirc and Acfs. 2

Supplementary Figure 2. CircFusion parameter tuning analysis on simulated RNA-seq datasets. 4

Supplementary Figure 3. Expression of circKMT2A and circAFF1 isoforms, and of f-circRNAs in BCP-ALL samples. 5

Supplementary Tables **6**

Supplementary Table 2. Genomic coordinates of the linear fusion breakpoints detected in acute lymphoblastic leukemia RNA-seq samples harboring the KMT2A::AFF1 translocation. 6

Supplementary Table 3. F-circRNAs detected by CircFusion in patient samples with KMT2A::AFF1 translocations. 7

References **7**

Supplementary Methods

Read alignment to the reference sequences

The STAR aligner v2.7.5 [1] was used to align RNA-seq reads to the fusion reference sequence set with the following parameters: outFilterMultimapNmax=10, outFilterMatchNmin=5, outFilterMismatchNmax=4, outFilterMismatchNoverReadLmax=0.01, alignSJstitchMismatchNmax=-1 -1 -1 -1.

The Samtools tool v1.10 [2] was used to remove duplicates in the alignment file and for BAM file indexing. The conversion of BAM files into BED format was performed with BEDtools v2.27.1 [3].

Supplementary Figures

Supplementary Figure 1. Precision and recall in simulated RNA-seq datasets of CircFusion, Fcirc and Acfs.

(A) Performance of f-circRNA and linear fusion transcripts detection (red, blue and green colors represent CircFusion (CF), Fcirc and Acfs, respectively; light and dark colors are used for 100 and 150 nt reads). The performance of the three methods are shown separately by (B) fusion type, (C) junction type, and (D) in presence or absence of translocations involving paralog genes (colors as in A; brp, breakpoint; fusion, fusion transcript; alt. fusion, alternative fusion transcript).

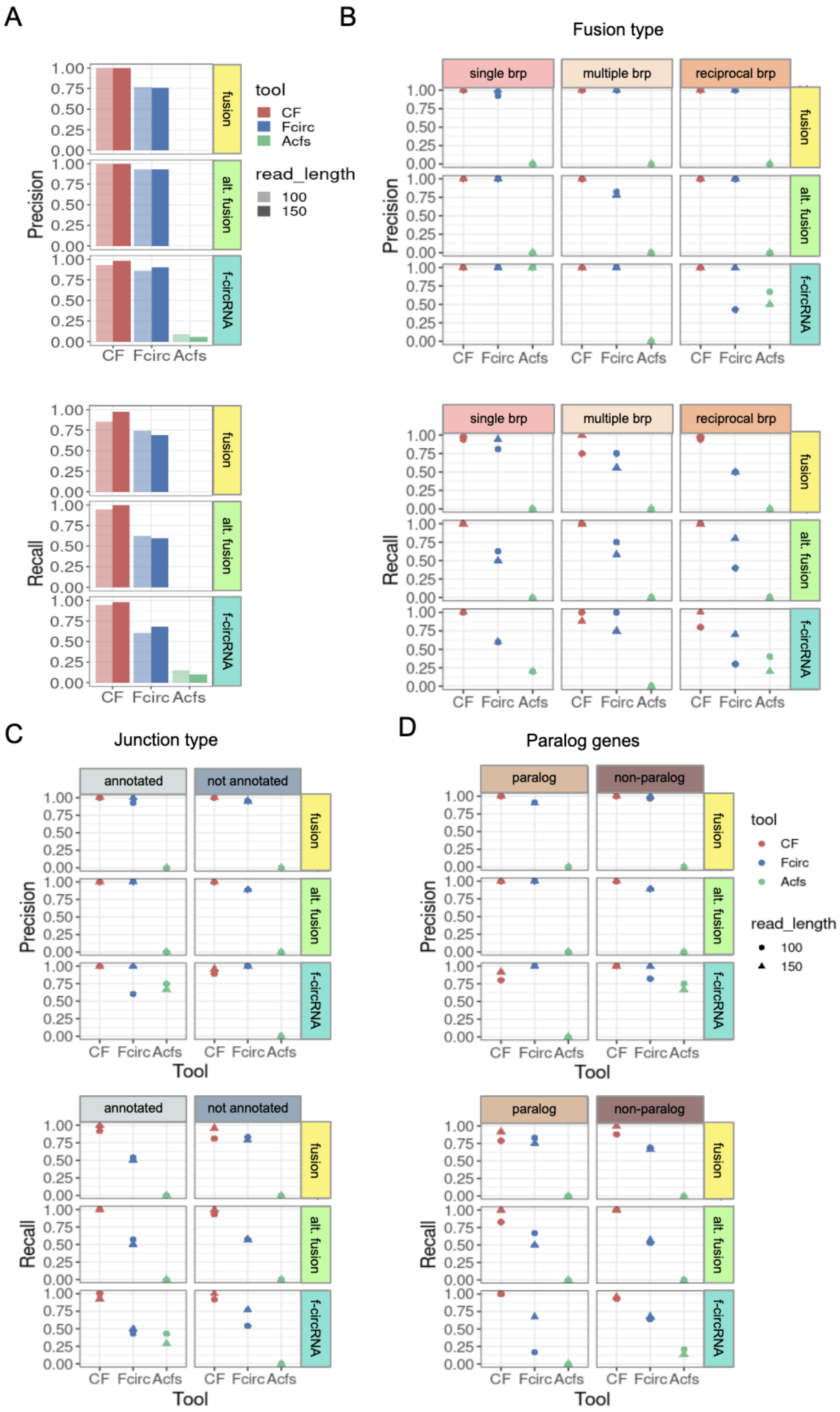
A
B
C
D
Fusion type
Junction type
Paralog genes
single brp
multiple brp
reciprocal brp
annotated
not annotated
paralog
non-paralog
fusion
alt. fusion
f-circRNA
Precision
Recall
1.00
0.75
0.50
0.25
0.00
CF
Fcirc
Acfs
Tool
tool
read_length
100
150

Supplementary Figure 2. CircFusion parameter tuning analysis on simulated RNA-seq datasets.

CircFusion Recall (A), precision (B) and F1 score (C) evaluated with combinations of different overlap values over the breakpoint junction (1 to 10, 15 and 20 nt) and minimal numbers of matches required in the alignment (lines with different shades of color), showing three kinds of investigated transcripts and two read lengths separately (fusion, fusion transcript; alt. fusion, alternative fusion transcript).

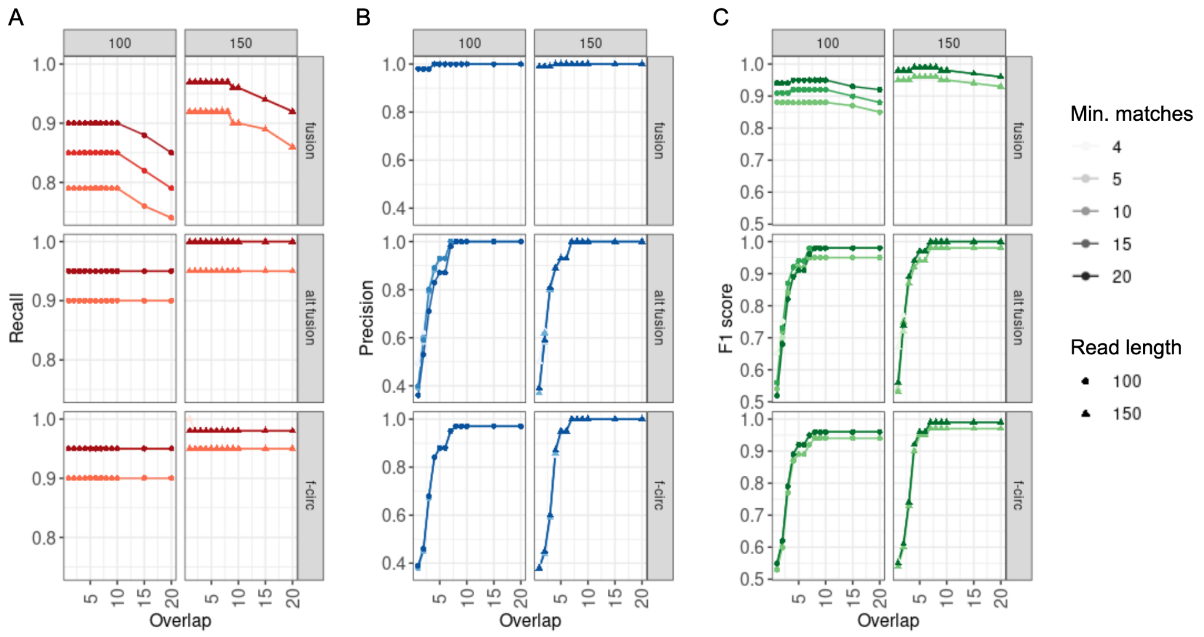

Supplementary Figure 3. Expression of circKMT2A and circAFF1 isoforms, and of f-circRNAs in BCP-ALL samples.

CircRNAs are ordered by median expression across samples where they are detected (cross symbol). F-circRNAs are identified by the gene exons backpliced (f.i. f-circA15-K17 is produced by backplicing AFF1 exon 15 with KMT2A exon 17), whereas canonical circRNAs are identified by letters as in [4] and by backpliced exons.

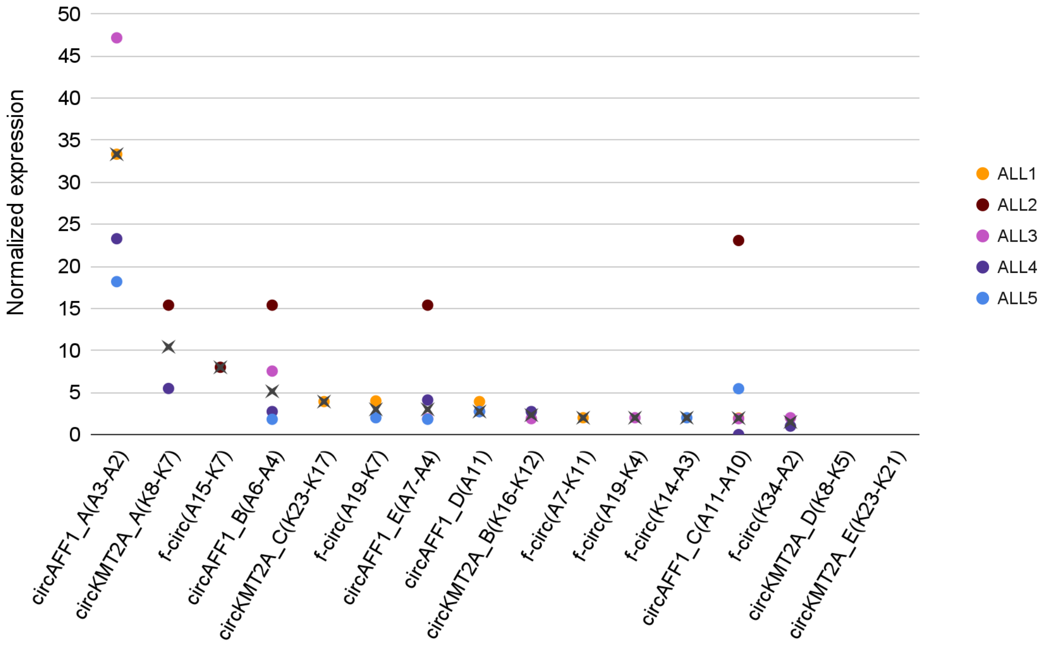

Supplementary Tables

Supplementary Table 2. Genomic coordinates of the linear fusion breakpoints detected in acute lymphoblastic leukemia RNA-seq samples harboring the *KMT2A::AFF1* translocation.

Exon numbers are according to the transcripts ENST00000389506 (*KMT2A*) and ENST00000307808 (*AFF1*) (Ensembl GRCh38 v.93).

Sample	Gen. coordinate KMT2A	Gen. coordinate AFF1	Breakpoint exon 5'	Breakpoint exon 3'	Fusion breakpoint
RS4;11	chr11: 118,484,314	chr4: 87,084,123	9	4	Direct 1
RS4;11	chr11: 118,482,495	chr4: 87,084,121	8	4	Direct 2
RS4;11	chr11: 118,484,309	chr4: 87,047,594	10	3	Reciprocal 1
RS4;11	chr11: 118,484,971	chr4: 87,047,594	11	3	Reciprocal 2
ALL1	chr11: 118,488,760	chr4: 87,089,984	11	5	Direct 1
ALL2	chr11: 118,482,495	chr4: 87,084,120	8	4	Direct 1
ALL3	chr11: 118,488,614	chr4: 87,084,163	10	5	Direct 1
ALL3	chr11: 118,484,863	chr4: 87,084,162	9	5	Direct 2
ALL3	chr11: 118,488,630	chr4: 87,084,164	11	4	Reciprocal 1
ALL3	chr11: 118,488,758	chr4: 87,084,164	12	4	Reciprocal 2
ALL4	chr11: 118,484,314	chr4: 87,047,590	9	4	Direct 1
ALL4	chr11: 118,488,758	chr4: 87,084,122	12	3	Reciprocal 1
ALL4	chr11: 118,484,973	chr4: 87,108,315	11	10	Reciprocal 2
ALL5	chr11: 118,482,092	chr4: 87,047,593	7	4	Direct 1
ALL5	chr11: 118,482,495	chr4: 87,047,593	8	4	Direct 2
ALL5	chr11: 118,488,759	chr4: 87,084,122	12	3	Reciprocal 1
ALL5	chr11: 118,488,630	chr4: 87,084,164	11	4	Reciprocal 2

Supplementary Table 3. F-circRNAs detected by CircFusion in patient samples with *KMT2A::AFF1* translocations.

Exon numbers are according to the transcripts ENST00000389506 (*KMT2A*) and ENST00000307808 (*AFF1*) (Ensembl GRCh38 v.93). The number of supporting reads is normalized over 10 M of linearly unmapped reads.

Sample	Fusion	Backspliced Exon 5'	Backspliced Exon 3'	F-circRNA	Reads
ALL1	AFF1::KMT2A	19	7	f-circA19-K7	4
ALL1	AFF1::KMT2A	7	11	f-circA7-K11	2
ALL2	AFF1::KMT2A	15	7	f-circA15-K7	8
ALL3	AFF1::KMT2A	19	4	f-circA19-K4	2
ALL3	KMT2A::AFF1	34	2	f-circK34-A2	2
ALL4	KMT2A::AFF1	34	2	f-circK34-A2	1
ALL5	AFF1::KMT2A	19	7	f-circA19-K7	2
ALL5	KMT2A::AFF1	14	3	f-circK14-A3	2

References

1. Dobin A, Davis CA, Schlesinger F, Drenkow J, Zaleski C, Jha S, et al. STAR: ultrafast universal RNA-seq aligner. Bioinformatics. 2013;29:15–21.

2. Li H, Handsaker B, Wysoker A, Fennell T, Ruan J, Homer N, et al. The Sequence Alignment/Map format and SAMtools. Bioinformatics. 2009;25:2078–9.

3. Quinlan AR. BEDTools: The Swiss-Army Tool for Genome Feature Analysis. Curr Protoc Bioinformatics. 2014;47:11.12.1–34.

4. Dal Molin A, Bresolin S, Gaffo E, Tretti C, Boldrin E, Meyer LH, et al. CircRNAs Are Here to Stay: A Perspective on the Recombinome. Front Genet. 2019;10:88.
